# 2,4-Epibrassinolide Mitigates Cd Stress by Enhancing Chloroplast Structural Remodeling and Chlorophyll Metabolism in *Vigna angularis* Leaves

**DOI:** 10.3390/biology14060674

**Published:** 2025-06-10

**Authors:** Suyu Chen, Zihan Tang, Jialin Hou, Jie Gao, Xin Li, Yuxian Zhang, Qiang Zhao

**Affiliations:** 1 Key Laboratory of Ministry of Agriculture and Rural Affairs of Soybean Mechanized Production, Heilongjiang Bayi Agricultural University, Daqing 163319, China; chensuyu999@163.com (S.C.); hjialin_27@163.com (J.H.); 13936979283@163.com (X.L.); 2 National Coarse Cereals Engineering Research Center, Daqing 163319, China; 3 Liaoning Key Laboratory of Urban Integrated Pest Management and Ecological Security, College of Life Science and Bioengineering, Shenyang University, Shenyang 110044, China; tangzhget@163.com; 4 Inner Mongolia Technology Promotion Center of Agricultural & Animal Husbandry, Hohhot 010031, China; gaojieflying@163.com

**Keywords:** antioxidant defense, chlorophyll metabolism, chlorophyll synthesis and decomposition, heavy metal stress, leaf microstructure

## Abstract

Cadmium (Cd) is a highly hazardous heavy metal that has an extensive impact throughout the world. 2,4-Epibrassinolide (BR) is an endogenous hormone that can enhance plant tolerance to various abiotic stresses. Herein, *Vigna angularis* cultivar “Zhen Zhuhong” was grown hydroponically and treated with 0, 1, and 2 mg·L^−1^ cadmium chloride (CdCl_2_) at the V1 stage, and foliar sprayed with or without 1 μM BR solution to analyze the effects of BR treatment on the physiology of *Vigna angularis* seedling leaves under Cd stress. BR treatment significantly alleviated the growth inhibition induced by Cd stress, which was associated with an increase in the plant height (11.15–17.83%), leaf area (35.59–56.72%), leaf dry weight (45.57–50.65%), and above-ground dry weight (50.86–55.17%). In addition, BR treatment induced significant reductions in Cd accumulation across different tissues of *V. angularis*, with decreases of 20.38–35.93% in leaves, 21.24–32.74% in stems, and 15.38–16.00% in petioles. Compared with the Cd treatment, BR treatment significantly enhanced the activities of peroxidase (5.02–13.22%), ascorbate peroxidase (27.13–70.28%), catalase (20.46–32.30%), and superoxide dismutase (16.54–21.81%), and increased the ascorbic acid content (27.55–45.52%), which contributed to a reduction in the accumulation of reactive oxygen species, cellular membrane damage, and cytoplasmic exosmosis. RNA-seq and real-time quantitative reverse transcription PCR analyses revealed that the BR treatment under Cd stress significantly upregulated the expression of genes involved in chlorophyll biosynthesis, transformation, and degradation, thereby enhancing the chlorophyll cycle. Furthermore, the BR treatment significantly increased the number of grana lamellae in the mesophyll cells, which enhanced the biosynthesis of chloroplasts. The increase in the chlorophyll content improved the capture of light energy, electron transport in photosynthesis, and the biosynthesis and metabolism of carbohydrates in the leaves of *V. angularis* under Cd stress.

## Simple Summary

Cadmium (Cd) is one of the most widely distributed toxic heavy metal pollutants worldwide, seriously affecting the survival of plants and animals. In this study, foliar spraying of 1 μM 2,4-epibrassinolide was shown to upregulate the expression levels of chlorophyll synthesis-related genes in adzuki bean (*Vigna angularis*) leaves, thereby increasing chlorophyll content and stabilizing chloroplast structure. These effects were beneficial for improving the photosynthetic capacity of the plants, providing more energy and material supply for plant stress resistance and growth. Higher cellular activity promoted the efflux and intracellular sequestration of Cd ions, reducing Cd accumulation in cells. The combined actions enhanced the Cd tolerance of adzuki bean plants. These results provide a theoretical basis for the cultivation of Cd-tolerant and low-Cd-content grain crops.

## 1. Introduction

Cadmium (Cd) is a highly hazardous heavy metal that has widespread effects throughout the world. It can be absorbed and accumulated by plant roots and then passed along the food chain into the human body, which ultimately adversely impacts human health [1,2,3]. Furthermore, Cd can directly induce various symptoms of damage in plants, such as the inhibition of photosynthesis, damage to DNA, oxidative stress, apoptosis, and cell cycle arrest, and therefore inhibits growth and results in a reduction in yield [4,5,6]. Thus, exploring the mechanisms of Cd toxicity in plants and cultivating crops that are tolerant to Cd and accumulate low levels of it are important to improve the yield of seeds and ensure food security.

Cd is not a required metallic element for plant growth. However, it can be absorbed by plant roots through membrane protein transporters intended for divalent metal ions, such as those of iron (Fe^2^⁺), manganese (Mn^2^⁺), zinc (Zn^2^⁺), and calcium (Ca^2^⁺) [3,7]. Cd can interact directly with the hydrogen bonds of base pairs in both genomic DNA and organellar DNA, such as those in mitochondria and chloroplasts. This leads to various types of reversible or irreversible DNA damage in plants [6,8]. These types of damage affect genome stability, disrupt protein biosynthesis, and ultimately cause disruptions in physiological metabolism [9]. Moreover, Cd stress can induce the accumulation of reactive oxygen species (ROS) in the mitochondria, chloroplasts, plasma membranes, the endoplasmic reticulum, and peroxisomes in plants, which triggers oxidative stress [10]. For example, Cd stress-induced ROS accumulation in cucumber (*Cucumis sativus*) can promote cytochrome c (Cyt c) release from mitochondria and increase mitochondrial permeability transition pore (MPTP) opening, leading to root tip cell death [11]. Chloroplast DNA (cpDNA) is highly susceptible to the damage caused by ROS owing to its lack of histone packaging, and its location near the source of ROS production. Furthermore, since both the nuclear DNA and cpDNA jointly determine the genetic expression of chloroplasts, the direct or indirect damage to nuclear DNA and cpDNA by Cd or ROS will also alter the structure and quantity of chloroplasts in plants [12,13]. Furthermore, Cd can directly substitute for metal ions, such as Ca^2+^ and Mg^2+^, within the chlorophyll molecule, thereby affecting the biosynthesis and stability of chlorophyll. This consequently impacts the photosynthetic capacity and supply of carbohydrates [14,15].

The first phenotypic changes in plants induced by abiotic stress are leaf chlorosis and wilting. Typically, the process of senescence in leaves begins with an imbalance in chlorophyll biosynthesis and degradation within the mesophyll cells [15]. Chlorophyll can be classified into four forms based on its chemical properties, namely chlorophyll *a* (*Chl a*), chlorophyll *b* (*Chl b*), chlorophyll *c* (*Chl c*), and chlorophyll *d* (*Chl d*) [16]. *Chl a* and *Chl b* are found in higher plants, whereas *Chl c* and *Chl d* are more abundant among photosynthetic algae [17,18,19]. In higher plants, *Chl a* is essential for the capture of light energy and its conversion in photosystem I (PSI) and photosystem II (PSII). *Chl b* primarily engages in the peripheral capture of light energy in both PSI and PSII [20]. The biosynthesis of chlorophyll is a highly orchestrated enzymatic process that is regulated by various enzymes encoded by 27 genes. Any alteration in these genes can disrupt the regulation of the enzymatic reactions, which subsequently impacts the biosynthesis of chlorophyll [21]. The degradation of chlorophyll primarily encompasses enzymatic and photodegradative pathways. The primary route for its degradation is enzymatic, and it involves the synergistic action of multiple key enzymes. *Chl b* undergoes reversible conversion to *Chl a*, which is facilitated by the enzymes *Chl b* reductase (CBR) and 7-hydroxymethyl-*Chl a* reductase (HCAR), through the intermediate 7-hydroxymethyl-*Chl a*. [22]. Pheide oxygenase (PAO) and red chlorophyll catabolite reductase (RCCR) convert Pheide a into nonfluorescent chlorophyll catabolites (NCCs) [23,24]. The chlorophyll metabolic cycle is instrumental in the regulation of plant growth and development, and it also helps to facilitate the acclimation of plants to environmental changes [20]. On the one hand, the chlorophyll cycle facilitates the redistribution of nutrients in plant leaves [25]. Alternatively, the products of chlorophyll degradation, such as Pheide, are photosensitive and may act as photoprotective compounds and mitigate the photooxidative damage caused by stress [26]. In addition, the stress caused by high concentrations of Cd can disrupt the structure of the mesophyll cells, which causes the disorganization of the chloroplast grana and thylakoid lamellae; this damages the integrity of the chloroplasts and decreases the chlorophyll content [27].

Abiotic stress also reduces the activity of the reaction centers of the photosystems, which inhibits photosynthetic electron transport and affects the absorption and conversion of light energy [13,28]. Additionally, Cd stress leads to a decrease in stomatal conductance in leaves, which reduces the pathways for the entry of carbon dioxide. This is accompanied by a decrease in the rate of transpiration [29]. Previous research has shown that Cd stress has a notable effect on rice (*Oryza sativa*), decreasing its net photosynthetic rate and light saturation point, while simultaneously elevating its light compensation point [30]. Plants that are subjected to Cd stress experience a decrease in their photosynthetic rate and the absorption of trace elements, such as zinc, manganese, iron, and copper, which inhibits the height and volume of the roots of *Pennisetum* plants [31]. Cd stress can significantly decrease the photosynthetic pigment content, transpiration rate, stomatal conductance, and net photosynthetic rate in the leaves of maize (*Zea mays*) [32].

Brassinosteroids (BRs) are important regulators in various plant processes, including cell division, xylem differentiation, and photomorphogenesis, while simultaneously aiding in the modulation of the responses of plants to abiotic stresses [33]. For example, BR treatment can enhance leaf photosynthesis by promoting the biosynthesis of chlorophyll and affecting the activity of photosynthetic enzymes of rice [34] and *Wolffia arrhiza* [35] under Cd stress. Furthermore, BR treatment also facilitates Cd sequestration in cell walls, thereby reducing its translocation to grains. Also, BR treatment has been proven to enhance the photosynthetic capacity of mung bean (*Vigna radiata*) under shading conditions by increasing photosynthetic enzyme activities, improving both the quantum yield of PSII electron transport and energy capture efficiency [36]. Additionally, regulating endogenous BR content in plants also enhances their resistance to abiotic stress [37]. However, studies on the effects of BR on the leaf structure and chlorophyll metabolism cycle of *Vigna angularis* under Cd stress are still lacking.

*V. angularis* is rich in flavonoids and is a coarse grain crop used both for food and medicine. With the continuous escalation of environmental pollution problems, the phenomenon of Cd stress during *V. angularis* production has become increasingly common. Therefore, establishing a cultivation measure resistant to Cd stress and producing low-Cd-accumulation grains has become one of the most important goals in coarse grain crop production. Herein, the *Vigna angularis* cultivar “Zhen Zhuhong” was used to evaluate the effect of BR treatment under Cd stress on the (1) growth and the accumulation of Cd; (2) ROS scavenging and osmotic adjustment; (3) chlorophyll metabolic cycle and photosynthetic regulation; and (4) remodeling of the structures of the mesophyll cells.

## 2. Materials and Methods

### 2.1. Materials, Growth, and Treatment Conditions

The seeds of *V. angularis* cultivar “Zhen Zhuhong” were provided by the Soybean Cultivation Innovation Team, College of Agronomy, Heilongjiang Bayi Agricultural University (Daqing, China). Average-sized seeds were sown in vermiculite and placed in a light incubator (photoperiod, 16/8 h (light/dark); temperature 26 ± 2 °C; humidity, 50–55%) for 5 days until the true leaves had spread.

Uniformly growing seedlings were selected and transferred to polypropylene pots (30 cm long, 20 cm wide, and 8.5 cm high) filled with 1/2 Hoagland’s nutrient solution (Yuanye, China) for hydroponic culture. Each pot had eight *V. angularis* seedlings. The Cd stress was carried out in the form of CdCl_2_ (Macklin Biochemical, Shanghai, China) when the first compound leaf of the *V. angularis* seedlings was fully displayed (10 days after transplanting). The CdCl_2_ was dissolved in the 1/2 Hoagland’s nutrient solution to prepare solutions with concentrations of 0 (Cd0), 1 (Cd1), and 2 (Cd2) mg·L^−1^. For the BR treatment, 10 mL of 1 μM 2,4-epibrassinolide (purity ≥ 92%, YuanYe, Shanghai, China) solution was foliar sprayed on each *V. angularis* seedling. A volume of 10 mL of deionized water was sprayed on the seedling leaves as the control treatment (CK). Both the BR solution and deionized water were mixed with 0.01% (*v*/*v*) of surfactant L-77 (Solarbio, Beijing, China). The seedlings were cultivated in the chamber for another 14 days. The third compound leaf was collected and flash frozen in liquid nitrogen for 5 min, then stored at −80 °C [13]. The eight plants in each pot were regarded as an experimental unit. Each treatment had three replicates.

### 2.2. Biomass and Morphological Measurements

The height of the plant was measured using a ruler from the cotyledonary node to the growing point of plant main stem. A leaf area meter (Yaxin-1241, Beijing Yaxinliyi Science and Technology, Beijing, China) was used to measure the total surface area of the leaves. The enzymes in the fresh tissue samples were deactivated by drying in a 105 °C oven (BGZ-246, Shanghai Boxun Medical Biological, Shanghai, China) for 30 min and then at 80 °C until they attained a stable weight. The dry weight of the samples was measured with a 0.0001 g precision balance. The rate of inhibition (%) on the growth of seedlings subjected to different concentrations of Cd stress was calculated as follows:

(1 − leaf area of treatment group/leaf area of control group) × 100%(1)

### 2.3. Cadmium (Cd) Content Measurement

The dry samples (0.25 g) were ground and dissolved in 5 mL of nitric acid (HNO_3_), 2 mL of HCl, 6 mL of perchloric acid (HClO_4_), and hydrogen peroxide (H_2_O_2_). The reaction solution was brought to a volume of 50 mL with deionized water. A volume of 2 mL of the reaction solution was diluted to 10 mL using 1% HNO_3_ (*v*/*v*) to measure the Cd content. The Cd standard solution was purchased from Solarbio (Cat. BYC1022, Solarbio). The concentration of Cd in the samples was quantified using an atomic absorption spectrometer (iCE 3500, Thermo Fisher Scientific, Waltham, MA, USA).

### 2.4. Photosynthetic Pigment Content and Chlorophyll Fluorescence Measurement

The photosynthetic pigments were extracted from 0.1 g of fresh leaves using 10 mL of 95% Ethanol. The light absorption values of the extraction solution at 470 nm, 649 nm, and 665 nm were determined using an absorbance microplate reader (SpectraMax 190, Molecular Devices, San Jose, CA, USA). The *Chl a*, *Chl b*, and carotenoids contents were calculated as described by Zhang [38].

A chlorophyll fluorescence analyzer (OS5p+, Opti-Sciences Inc., Hudson, NH, USA) was used to measure the parameters for chlorophyll fluorescence. The fluorescence intensities *F* and *F*_m′_ of the leaves were measured before and after treatment with saturation pulses in the light. The minimum fluorescence *(F*_o_) and maximum fluorescence (*F*_m_) values of the leaves were measured before and after a saturation pulse treatment on plants that had been in the dark for 30 min. The chlorophyll fluorescence parameters were calculated as follows:

PSII maximum photosynthetic efficiency

(*F*_v_/*F*_m_) = (*F*_m_ − *F*_o_)/*F*_m_ (2)

actual photosynthetic efficiency

(*Φ*_SPII_) = (*F*_m′_ − *F*)/*F*_m′_ (3)

minimum fluorescence value under light

*F*_o′_ = *F*_o_/((*F*_m_ − *F*_o_)/*F*_m_ + *F*_o_/*F*_m_) (4)

photochemical quenching coefficient

(qL) = (*F*_m′_ − *F*)/(*F*_m′_ − *F*_o′_) − *F*_o′_/*F* (5)

non-photochemical quenching coefficient

(NPQ) = *F*_m_/*F*_m′_ − 1 (6)

quantum yield of non-regulatory energy dissipation

(*Φ*_NO_) = 1/(NPQ + 1 + qL (*F*_m_/*F*_o_ − 1)) (7)

quantum yield of regulatory energy dissipation

(*Φ*_NPQ_) = 1 − *Φ*_PSII_ − *Φ*_NO_ (8)

### 2.5. Determination of Leaf Microstructure

Fresh leaf samples (1 × 1 cm) were cut using a hole punch and immediately transferred to a 2 mL tube filled with electron microscope fixative solution (Cat. G1102, Wuhan Servicebio Technology Co., Wuhan, China). The samples were kept at 25 °C for another 2 h and then stored at 4 °C for 48 h. A volume of 1% osmic acid was used to fix the samples for 5 h at 20 °C. The sections were dehydrated, permeated, embedded, cut, and stained as described by Zhao [13]. The cellular morphology was observed using a transmission electron microscope (HT7700, Hitachi, Tokyo, Japan). In addition, images were also collected during the observation.

### 2.6. ROS, MDA, and Antioxidant Contents and the Activity of Antioxidant Enzymes

Crude enzyme extracts were prepared by homogenizing 0.2 g of cryopreserved (−80 °C) fresh tissue in 2 mL precooled 50 mM phosphate buffered saline (PBS, pH 7.8). The homogenate was centrifuged at 12,000× *g* for 20 min at 4 °C, and the resulting supernatant was collected for analysis of the superoxide dismutase (SOD, EC 1.15.1.1), peroxidase (POD, EC 1.11.1.7), catalase (CAT, EC 1.11.1.6), and ascorbate peroxidase (APX, EC 1.11.1.11) activities, and the malondialdehyde (MDA) and superoxide anion (O_2_^−^) content following the description of Gao [39]. The POD activity was assayed using guaiacol, SOD was assayed using the nitrogen blue tetrazole technique, the CAT and APX were assayed using UV spectrophotometry at wavelengths of 240 nm and 290 nm, respectively. The O_2_^−^ content was determined using the hydroxylamine oxidation method, and the MDA content was determined using the thiobarbituric acid method.

The H_2_O_2_ and ascorbic acid (ASA) contents were determined using the potassium iodide method as described by Zhang [38]. The glutathione (GSH) and oxidized glutathione (GSSG) contents were measured using assay kits (Cat.BC1175 and BC1185, Solarbio).

### 2.7. Osmoregulatory Substance Content

The soluble proteins content was determined using the Bradford method with Coomassie Brilliant Blue G-250 and a standard of bovine serum protein, while the proline content was assayed using the sulfosalicylic acid method as described by Gao [39]. The free amino acids content was determined by the ninhydrin method as described by Li [40].

A total of 0.2 g of fresh leaves were immersed in 10 mL of deionized water for a duration of 24 h, followed by measurement of the initial electrical conductivity (R1). Subsequently, the electrical conductivity (R2) was measured after the solution had boiled for 10 min and cooled to room temperature. The relative conductivity (REC) of the leaves was determined using the following formula:

R1/R2 × 100% (9)

All the experiments were conducted in triplicate.

### 2.8. RNA Extraction, First-Strand cDNA Synthesis, and qRT-PCR Analysis

Total RNA from the leaves was extracted using a TransZol Plant reagent kit (Cat. ET121, Tiangen Biotech (Beijing), Beijing, China). The concentration of RNA was measured using a NanoDrop OneC (Thermo Fisher Scientific), and its integrity was examined by 1% agarose gel electrophoresis. Single-stranded cDNA was synthesized using the PerfectStart^®^ (Beijing, China) Uni RT & qPCR Kit (Cat. AUQ, TRAN). Each 20 µL reaction mixture contained 1.5 µg of total RNA.

The reaction mixture was diluted 8-fold before it was utilized for real-time quantitative reverse transcription PCR (qRT-PCR). Subsequently, 1 μL of the diluted cDNA was placed in a 20 μL reaction tube, and the qRT-PCR detection tests were conducted using the TransScript^®^ (Beijing, China) Top Green qPCR SuperMix Kit (Cat. AQ131, TRAN) on an ABI Step One™ Plus Real-Time PCR System (Thermo Fisher Scientific) [13]. The *V. angularis VaTUA3* gene was used as the internal control. The list of primers employed for qRT-PCR is provided in Table S1. The relative levels of gene expression among the various treatments were determined using the operational formula 2^−ΔΔCT^ [41]. The qRT-PCR experiments comprised three biological replicates, with each biological replicate consisting of three technical replicates.

### 2.9. RNA-seq Analysis

TRIzol (Invitrogen, Waltham, MA, USA) was used to extract the RNA from the leaves of *V. angularis* seedlings subjected to the Cd2 and BR + Cd2 treatments. Each treatment of the RNA-seq analysis had three biological replicates. The integrity and content of RNA were verified using an Agilent Biological Analyzer 2100 (Agilent Technologies, Palo Alto, CA, USA). The cDNA was synthesized using Invitrogen SuperScript^TM^ II reverse transcriptase (Cat. 18064071, Invitrogen). The cDNA was double-ended sequenced in the PE150 mode on an Illumina NovaSeq^TM^ 6000 (LC Bio Technology Co., Ltd., Hangzhou, China) as previously described. The low-quality reads, including sequencing joints, low-quality sequences, and other associated low-quality components, were removed using Cutadapt to obtain clean data to compare with the reference genome. The transcript was reconstructed using StringTie, followed by computation of the levels of expression for all the genes present in each sample. The differentially expressed genes (DEGs) were identified based on the following threshold criteria: |1og_2_ Fold Change| > 1 and q < 0.05. The RNA-seq results were checked by a real-time quantitative PCR (qPCR) analysis. The Gene Ontology (GO) and Kyoto Encyclopedia of Genes and Genomes (KEGG) enrichment of the DEGs between different treatments were analyzed using the cloud platform of LC Bio Technology Co., Ltd. (Hangzhou, China, www.omicstudio.cn, accessed on 1 November 2024).

### 2.10. Statistical Analysis

The measurement data of each treatment contained three biological replicates, and each biological replicate contained three technical replicates. The data were analyzed using SPSS 19.0 (IBM, Armonk, NY, USA), and the results are presented as the mean ± SD. One-way analysis of variance (ANOVA) was performed to evaluate the significant differences between the treatments at *p* < 0.05 level.

## 3. Results

### 3.1. BR Treatment Alleviates the Inhibition of V. angularis growth Under Cd Stress

**Figure 1:**
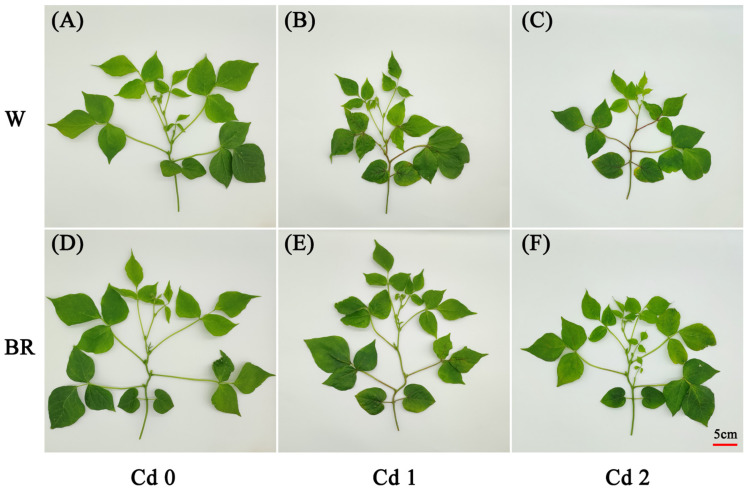
The effect of BR treatment on *V. angularis* growth under Cd stress. The plants sprayed with water under 0 mg·L^−1^ (**A**), 1 mg·L^−1^ (**B**) and 2 mg·L^−1^ (**C**) Cd stress treatments; the plants sprayed with 1μM BR under 0 mg·L^−1^ (**D**), 1 mg·L^−1^ (**E**), and 2 mg·L^−1^ (**F**) Cd stress treatments.

To evaluate the effect of BR treatment on the growth of *V. angularis* under Cd stress, the biomass and morphological parameters were measured after 7 days of different concentrations of Cd stress. When compared with the CK (W + Cd0), the 1 mg·L^−1^ (Cd1) and 2 mg·L^−1^ (Cd2) Cd stress treatments without the BR treatment significantly decreased the height, leaf area, leaf fresh wight, leaf dry wight and above-ground dry weight (Figure 1 and Table 1). The exogenous foliar spray of 1 μM BR significantly alleviated the inhibition of the growth of the *V. angularis* plants induced by Cd stress. Furthermore, there was no significant difference in the growth of the plants between the BR treatments and the CK.

To assess the effect of the BR treatment on the bioaccumulation of Cd in the *V. angularis* tissue, its content in the leaves, petiole, and stems were measured after 7 days of different concentrations of Cd. Compared with the CK, the Cd ions accumulated to significant levels in the leaves, stems and petioles of the seedlings and showed a significant dose dependence on the concentration of Cd (Table 1). The leaves accumulated the most Cd, while the petioles accumulated the least. In contrast to the Cd stress treatments, the application of BR significantly decreased the accumulation of Cd in the leaves, stems, and petioles.

### 3.2. BR Treatment Reduced the Oxidative Damage Induced by Cd in the V. angularis Leaves

**Figure 2:**
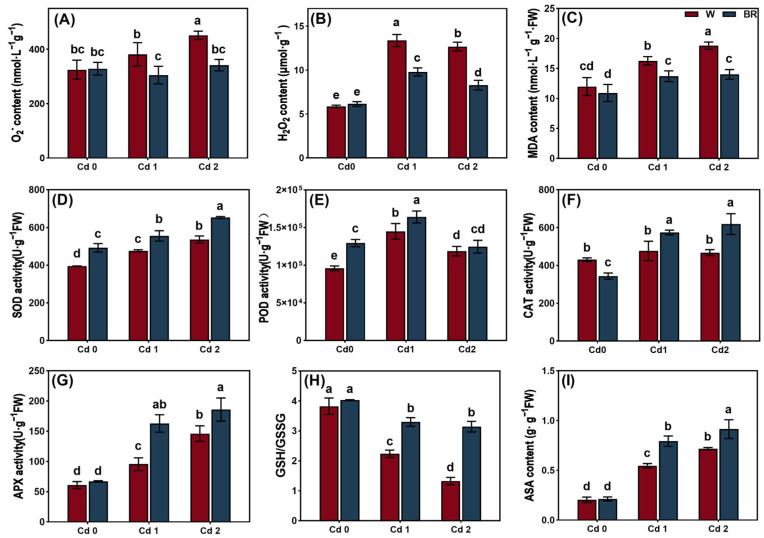
The effect of BR treatment on antioxidant defense of *V. angularis* seedling leaves under Cd stress. The O_2_^−^ (**A**), H_2_O_2_ (**B**), and MDA (**C**) content. The SOD (**D**), POD (**E**), CAT (**F**), and APX (**G**) activities. The GSH/GSSG value (**H**) and ASA content (**I**). Statistically significant differences (*p* < 0.05) among the various treatments are denoted by different letters.

To assess the effect of the BR treatment on the antioxidant defenses of the leaves under Cd stress, the ROS, MDA, and antioxidant contents were measured, and the antioxidant enzymes were assayed. The Cd stress treatments significantly increased the superoxide anion (O_2_^−^), H_2_O_2_, and MDA contents in the leaves when compared with the CK (Figure 2). The BR treatment significantly decreased the O_2_^−^ and MDA contents under Cd stress. It is notable that the BR treatment decreased the H_2_O_2_ content in the leaves under Cd1 stress but increased it in the leaves under Cd2 stress.

The Cd stress treatments significantly enhanced the activities of the antioxidant enzymes, including SOD, POD, and APX, in the leaves when compared with the CK. Furthermore, the Cd stress treatments increased the ASA content in the leaves. The BR treatment enhanced the activities of the antioxidant enzymes and the ASA content in the *V. angularis* leaves under Cd stress. Additionally, the Cd treatment decreased the GSH/GSSG value dose-dependently. However, the BR treatment reversed these effects by elevating the GSH/GSSG value under the Cd stress.

### 3.3. BR Treatment Regulated the Osmotic Regulation of V. angularis Leaves Under Cd Stress

**Figure 3:**
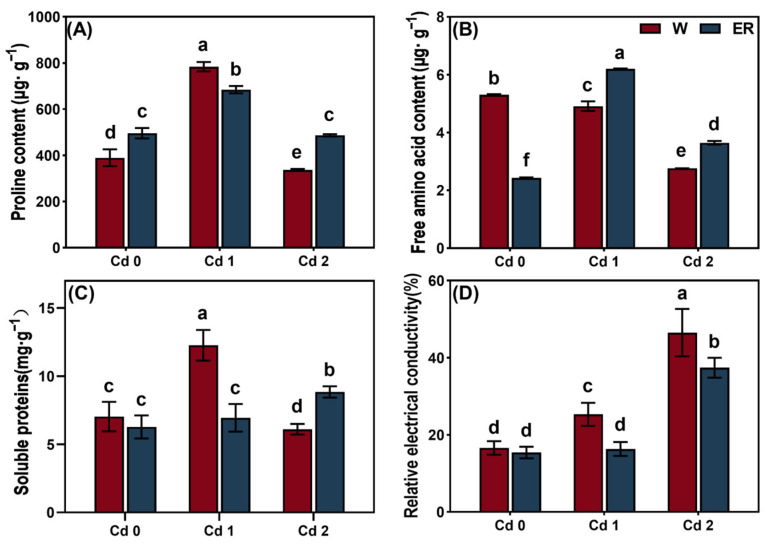
The effect of BR treatment on osmotic regulation of *V. angularis* seedling leaves under Cd stress. The proline (**A**), free amino acid (**B**), and soluble protein (**C**) content. The relative electrical conductivity (**D**) of *V. angularis* seedling leaves. Statistically significant differences (*p* < 0.05) among the various treatments are denoted by different letters.

To assess how the BR treatment influenced the osmotic adjustment mechanisms in the leaves when exposed to Cd stress, the proline, free amino acids, and soluble protein contents and the relative electrical conductivity (REC) were measured. Cd1 stress significantly increased the proline and soluble protein contents and the REC in the leaves when compared with the CK (Figure 3). Although the Cd2 stress decreased the proline, free amino acids, and soluble protein contents in the leaves, it increased the value of the REC. The BR treatment decreased the proline and soluble protein contents and the REC in the leaves under Cd1 stress, but it increased the free amino acid content. In addition, the BR treatment increased the proline, free amino acids, and soluble protein content in the leaves under Cd2 stress but deceased the REC.

### 3.4. BR Treatment Regulated Chlorophyll Content and Chlorophyll Fluorescence of V. angularis Leaves Under Cd Stress

**Figure 4:**
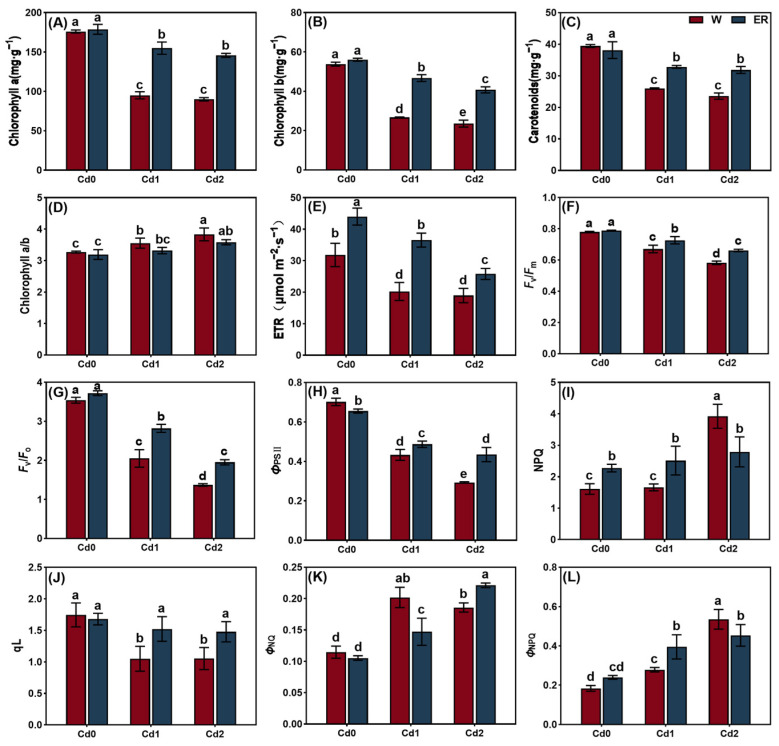
The effect of BR treatment on chlorophyll content and chlorophyll fluorescence of *V. angularis* seedling leaves under Cd stress. The *Chl a* (**A**), *Chl b* (**B**), and carotenoid (**C**) content in *V. angularis* seedling leaves. *Chl a/b* value (**D**), electron transport rate (ETR) (**E**), *F*_v_/*F*_m_ (**F**), *F*_v_/*F*_o_ (**G**), *Φ*_SPII_ (**H**), NPQ (**I**), qL (**J**), *Φ*_NO_ (**K**), and *Φ*_NPQ_ (**L**) in *V. angularis* seedling leaves. Statistically significant differences (*p* < 0.05) among the various treatments are denoted by different letters.

The chlorophyll content and chlorophyll fluorescence of the leaves were measured to evaluate the effect of the BR treatment on the chlorophyll metabolism and photosynthetic capacity of *V. angularis* leaves under Cd stress. The Cd stress treatments significantly decreased the *Chl a*, *Chl b*, and carotenoid contents compared with the CK (Figure 4). As expected, the BR treatment increased the photosynthetic pigment content compared with the CK under Cd stress but had no significant difference on the value for *Chl a/b*. In addition, the BR treatment significantly increased the *Chl b* content in the leaves compared with the water treatment under normal culture conditions. However, it had no significant effects on the *Chl a* and carotenoid contents.

In contrast to the CK, the Cd stress treatments significantly reduced the electron transport rate (ETR), *F*_v_/*F*_m_, *F*_v_/*F*_o_, *Φ*_SPII_, and qL in the leaves but enhanced the nonphotochemical chlorophyll fluorescence quenching (NPQ), *Φ*_NO_, and *Φ*_NPQ_. However, the BR treatment enhanced the ETR, *F*_v_/*F*_m_, *F*_v_/*F*_o_, *Φ*_SPII_, and qL in the *V. angularis* leaves under the stress of Cd1 and Cd2. In addition, the BR treatment enhanced the NPQ and *Φ*_NPQ_ in the leaves under Cd1 stress but reduced these values in the leaves under Cd2 stress. In contrast, the BR treatment reduced the *Φ*_NO_ in the leaves under Cd1 stress but enhanced the *Φ*_NO_ in the leaves under Cd2 stress.

### 3.5. BR Treatment Improved Microstructure of V. angularis Leaves Under Cd Stress

**Figure 5:**
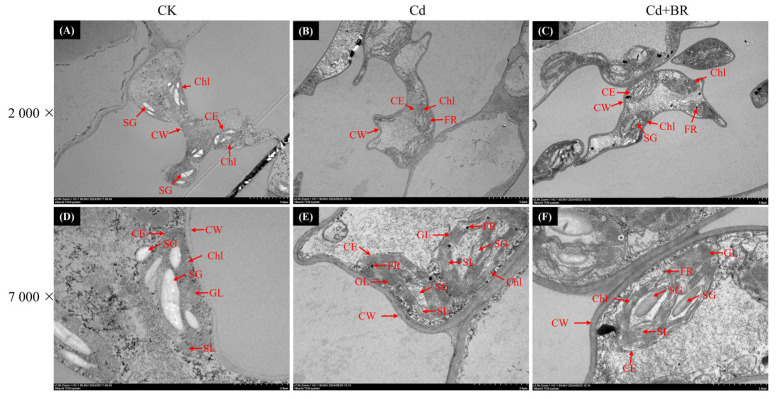
The effect of BR treatment on *V. angularis* leaf microstructure under Cd stress. The microstructure of *V. angularis* leaves under normal (**A**,**D**), Cd2 (**B**,**E**), and Cd2 + BR (**C**,**F**) treatment. CE refers to chloroplast envelope; SG refers to starch granule; GL refers to grana lamella; SL refers to stroma lamella; FR refers to free ribosomes.

To study the effect of the BR treatment on the microstructure of the *V. angularis* leaves under Cd stress, the microstructures of the mesophyll cells and chloroplasts were observed. The Cd stress treatments significantly reduced the cell volume but increased the cell wall thickness when compared with the CK (Figure 5). In addition, the Cd stress treatments significantly reduced the numbers of starch granules and grana lamellae but increased the numbers of stroma lamellae and free ribosomes. Compared with the Cd stress treatments, the BR treatment significantly increased the numbers of starch granules and grana lamellae in the mesophyll cells but decreased the numbers of stroma lamellae and free ribosomes.

### 3.6. Effect of BR Treatment on Transcription of Genes in V. angularis Leaves Under Cd Stress

**Figure 6:**
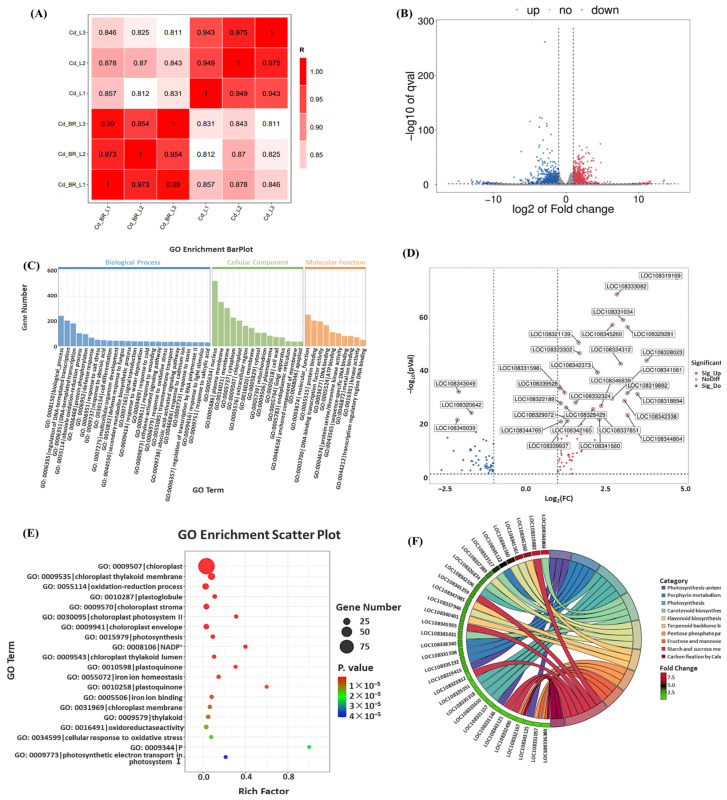
Effect of BR treatment on gene transcription in *V. angularis* leaves under Cd stress. Pearson correlation between three biological replicates under Cd2 or BR + Cd2 treatments (**A**); statistics of differentially expressed genes, where blue represents downregulated unigenes, and red represents upregulated unigenes (**B**); all DEGs enriched in GO terms (**C**); statistics of differentially expressed genes related to chloroplasts and photosynthetic terms, where blue represents downregulated unigenes, and red represents upregulated unigenes (**D**); a total of 122 DEGs enriched in GO terms (**E**); and the category of 122 DEGs (**F**).

RNA-seq was performed to evaluate the effect of the BR treatment on the expression of genes in the *V. angularis* leaves under Cd stress. The Pearson correlation between the three biological replicates under the Cd2 or BR + Cd2 treatments was >94% (Figure 6A), which indicated that the samples were highly repeatable, and the test was reliable. There were 936 upregulated (red) and 1266 downregulated (blue) genes in the leaves under the BR + Cd treatment compared with the Cd treatment (Figure 6B). The GO analysis results showed that the DEGs were significantly enriched in 84 terms. The top five significantly enriched terms in biological process were biological process (GO: 0008150), regulation of DNA-templated transcription (GO: 0006355), DNA-templated transcription (GO: 0006351), obsolete oxidation-reduction process (GO: 0055114), and protein phosphorylation (GO: 0006468). The top five significantly enriched terms in cellular component were nucleus (GO: 0005634), plasma membrane (GO: 0005886), membrane (GO: 0016021), cytoplasm (GO: 0005737), and chloroplast (GO: 0009507). The top five significantly enriched terms in molecular function were molecular function (GO: 0003674), protein binding (GO: 0005515), DNA-binding transcription factor activity (GO: 0003700), DNA binding (GO: 0003677), and ATP binding (GO: 0005524) (Figure 6C). A total of 122 DEGs related to chloroplasts and photosynthetic terms were screened by the RNA-seq experiment. These DRGs were related to the chloroplast (GO: 0009507), chloroplast thylakoid membrane (GO: 0009535), oxidation-reduction process (GO:0055114), plastoglobule (GO: 0010287), and chloroplast stroma (GO: 0009570) terms. Furthermore, the DEGs were also related to the photosynthesis antenna proteins (ko00196), porphyrin metabolism (ko00860), photosynthesis (ko00195), carotenoid biosynthesis (ko00906), and flavonoid biosynthesis (ko00941), among others (Figure 6D–F).

### 3.7. The BR Treatment Regulated the Photosynthetic Capacity of V. angularis Leaves Under Cd Stress

**Figure 7:**
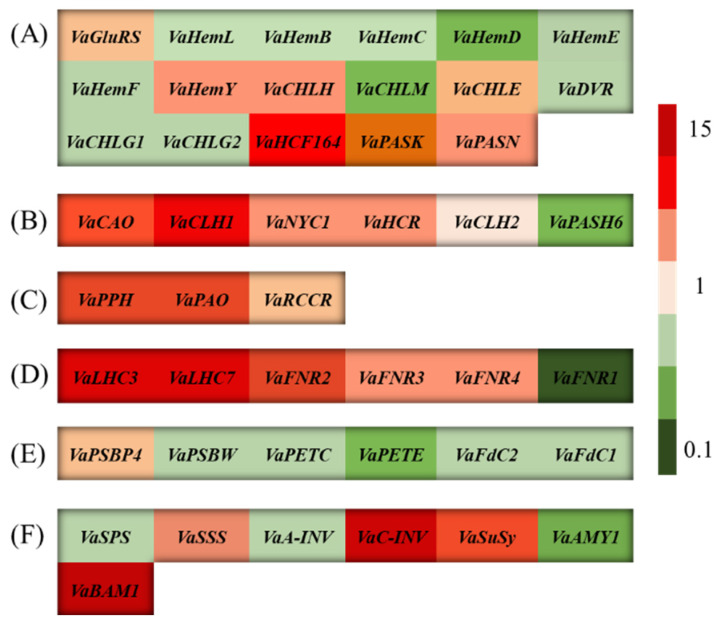
Effect of BR treatment on the photosynthetic capacity of *V. angularis* leaves under Cd stress. The relative expression levels of genes which participate in chlorophyll synthesis (**A**), the mutual transformation of *Chl a* and *Chl b* (**B**), chlorophyll degradation (**C**), light energy capture and electron transport in photosynthesis (**D**), the optical response pathway regulation in photosystem II (**E**), and carbohydrate synthesis and metabolism (**F**). The qRT-PCR analysis employed the operational formula 2^−ΔΔCT^, with the gene expression levels in *V. angularis* leaves subjected to Cd treatment serving as the baseline normalization value of 1. Data were collected from three independent experiments, and each biological replicate had three technical assays. Red represents genes upregulated, and green represents genes downregulated. The darker the color, the larger the change in the gene relative expression levels.

The levels of expression of the genes involved in chlorophyll biosynthesis, the mutual transformation of *Chl a* and *Chl b*, chlorophyll degradation, light energy capture and electron transport in photosynthesis, the optical response pathway regulatory genes in photosystem II, and the carbohydrate biosynthesis and metabolism were measured using qRT-PCR to examine the influence of the BR treatment on the photosynthetic capacity of the *V. angularis* leaves under Cd stress (Figure 7). Compared with the Cd stress treatments, the BR treatment significantly upregulated the gene expression levels of *VaGluRS*, *VaHemY*, *VaCHLH*, *VaCHLE*, *VaHCF164*, *VaPASK*, and *VaPASN*, which participate in the chlorophyll biosynthetic pathway, but downregulated the gene expression levels of *VaHemL*, *VaHemB*, *VaHemC*, *VaHemD*, *VaHemE*, *VaHemF*, V*aCHLM*, *VaDVR*, *VaCHLG1*, and *VaCHLG2* in the same pathway. The BR treatment also significantly upregulated the gene expression levels of the *VaCAO*, *VaCLH1*, *VaNYC1*, *VaHCR*, and *VaCLH2* genes involved in the mutual transformation of *Chl a* and *Chl b*, and *VaPPH*, *VaPAO*, and *VaRCCR* involved in the degradation of chlorophyll. Furthermore, the gene expression levels of the genes involved in the light energy capture and electron transport in photosynthesis, including *VaLHC3*, *VaLHC7*, *VaFNR2*, *VaFNR3*, and *VaFNR4*, and carbohydrate synthesis and metabolism, including *VaSSS*, *VaC-INV*, *VaSuSy*, and *VaBAM1*, in the *V. angularis* leaves were upregulated by the BR treatment. Conversely, the BR treatment downregulated the gene expression levels of *VaPSBW*, *VaPETC*, *VaPETE*, and *VaFdC2* involved in regulation of the photoresponse pathway in photosystem II (PSII) when comparted with the Cd treatment.

## 4. Discussion

Cadmium (Cd) absorbed by roots can affect the growth and development of plants by causing oxidative stress, which destroys the structure and function of biological macromolecules, and disrupts the structural stability of genomic DNA [42]. Generally, the phenotypic characteristics of plants under Cd stress are usually dwarfing, delayed growth and development, chlorotic leaves, and shortened stems [43,44]. Furthermore, Cd stress can inhibit physiological processes, such as photosynthesis and respiration, disrupt the balance of physiological metabolism, and suppress the accumulation of effective biomass [44]. In this study, the Cd stress treatments notably impeded the growth and accumulation of biomass in the *V. angularis* plants (Figure 1 and Table 1). Notably, the BR treatment significantly alleviated the inhibition of growth in the seedlings induced by Cd stress. Furthermore, the BR treatment significantly reduced the accumulation of Cd in the leaves, stems, and petioles. The results were consistent with previous studies which showed that treatment with BR can improve the tolerance of plants to abiotic stresses, such as cold, drought, and salinity [45,46,47].

In normal conditions, various types of ROS, such as peroxides, superoxide anions, hydroxyl radicals, singlet oxygen, and α-oxygen, are naturally occurring byproducts of redox metabolism within organisms and play pivotal roles in the processes of cellular signaling and the maintenance of homeostasis [13]. However, abiotic stresses, such as high temperature, drought, salinity, metal toxicity, and UV-B radiation, can dramatically increase the ROS content in plants and lead to oxidative stress [11]. In mitochondria, a portion of O_2_ is reduced to ROS when the electrons are transferred to O_2_, and NADPH oxidase can catalyze the production of ROS on the plasma membranes of cells; it is also an important source of ROS [48,49]. In chloroplasts, the excess light energy absorbed by the photosynthetic pigments can stimulate the electron transport chain in the chloroplast, which leads to the production of singlet oxygen (^1^O_2_) and superoxide anions (O_2_^−^), and further increases the content of H_2_O_2_ [20]. Furthermore, some enzymatic reactions and flavin dehydrogenase in the electron transport processes in PSI and PSII are the primary pathways for the production of ROS in chloroplasts [50]. Plants can maintain the balance of cellular ROS through their intricate system of antioxidants, which is composed of antioxidant enzymes, such as SOD, POD, CAT, and APX, and non-enzymatic antioxidants, such as GSH and vitamins C/E, thereby balancing the production of ROS [51]. Previous research results showed that BR treatment enhanced tolerance to salt stress in cowpea (*Vigna sinensis*) by inducing enhanced activities of antioxidant enzymes such as SOD, POD, polyphenol oxidase (PPO), as well as increased ascorbic acid and glutathione contents [52]. Consistent results were obtained in Cd-exposed mung bean, where BRs ameliorated Cd-induced stress through augmentation of the antioxidant defense system [53]. Consistent with previous research, this study showed that the BR treatment significantly enhanced the activities of antioxidant enzymes, including POD, CAT, APX, and SOD, and the non-enzymatic antioxidant contents, including GSH and ASA, in the leaves of *V. angularis* under Cd stress, while reducing the H_2_O_2_, O_2_^−^ and MDA contents (Figure 2).

Abiotic stresses can also disrupt the balance of intracellular and extracellular water [30,54]. Osmotic stress can induce NADPH oxidase to aggregate on the cell plasma membrane by activating the Rho of plant proteins (ROP) and form the ROS-producing complex, thus triggering the accumulation of ROS and downstream physiological responses [55,56]. As one of the earliest cellular responses to osmotic stress, ROS signaling can regulate the downstream physiological processes, such as endocytosis, the absorption of water, and the accumulation of proline, which help the plants to respond to osmotic stress, maintain cell homeostasis, and survive quickly and accurately [57]. In this study, the Cd1 treatment significantly increased the proline and soluble protein contents and the REC in *V. angularis* leaves. However, the Cd2 treatment reduced the proline, free amino acids, and soluble proteins contents in the leaf cells (Figure 3). The results could be because the *V. angularis* can improve the intracellular osmotic potential by actively accumulating various organic or inorganic substances to respond to the low concentrations of Cd stress that induce osmotic and oxidative stress. Conversely, a high concentration of Cd stress can seriously damage the cell membrane, which results in the leakage of cell contents, and increases the relative electrical conductivity of *V. angularis*. In addition, under high-concentration Cd stress, plants shift from “active defense” to “passive damage” [58,59]. At this point, photosynthetic failure leads to disruption of sugar supply, inactivation of enzyme systems inhibits the synthesis of osmoregulatory substances, and the collapse of antioxidant mechanisms causes excessive consumption of reactive oxygen species (ROS) or loss of detection signals, resulting in irregular changes in the content of cellular components [60,61]. This phenomenon is usually accompanied by increased cell membrane permeability (such as elevated electrical conductivity) and activation of programmed cell death (PCD), serving as a marker of plants’ inability to tolerate extreme stress [62].

As expected, the BR treatment participated in the osmotic adjustment of the leaves under Cd stress, which mitigated the extent of the damage to the cell membrane.

Abiotic stress can reduce the capacity of plants to photosynthesize by limiting the stomata and non-stomata [63]. Stomatal restriction serves as a crucial regulatory mechanism for plants to manage short-term stress by decreasing the conductance of the stomata, thereby reducing the influx of carbon into the leaves. Non-stomatal limitations include a decrease in the photosynthetic rate caused by factors such as a reduction of leaf area, structural damage to the photosynthetic organs, and decreased activity of the enzymes involved in photosynthesis. Previous research has indicated that abiotic stress can disrupt the metabolism of chlorophyll in plants, inhibit the biosynthesis of aminolevulinic acid (ALA) into *Chl a*, and cause symptoms such as the loss of chlorophyll, senescence, and wilting. For example, high temperature stress significantly downregulates the level of expression of the gene that encodes porphobilinogen deaminase (*PBGD*) in tomato (*Solanum lycopersicum*) leaves but upregulates the level of expression of the *CHLASE* gene that encodes chlorophyllase, which accelerates the degradation of chlorophyll [64]. Salt stress significantly increases the level of expression of the gene that encodes the chlorophyll-degrading enzyme (PPH) in apple (*Malus halliana* and *Malus robusta*) rootstocks, which leads to the suppression of chlorophyll biosynthesis [65]. Cd stress significantly decreased the *Chl a*, *Chl b*, and carotenoid contents in the *V. angularis* leaves in this study (Figure 4). Furthermore, Cd stress significantly reduced the ETR, *F*_v_/*F*_m_, *Φ*_PSII_, and qL in the leaves, while significantly increasing NPQ. These results indicate that the Cd stress damaged the photosystem reaction centers in the leaves, which impeded the photosynthetic electron transport. In addition, there was an increase in the thermal dissipation of the leaves to mitigate the strong light damage that occurs under Cd stress. Notably, Cd stress significantly reduces the sizes of mesophyll cells and the numbers of starch grains and granule lamellae in the leaves, while increasing the number of stroma lamellae and free ribosomes (Figure 5). These results indicate that Cd stress significantly affects the structure of the chloroplast, which leads to a decrease in the chlorophyll content. As expected, the BR treatment can reshape the structure of mesophyll cells and chloroplasts under Cd stress, which enhances the chlorophyll content and improves the plant’s photosynthetic capacity and accumulation of carbohydrates. Concurrently, the results of RNA-seq and qRT-PCR demonstrate that the BR treatment significantly upregulated the levels of expression of the key genes involved in the biosynthesis of *Chl a*, conversion of *Chl a* to *Chl b*, and the chlorophyll degradation pathways in the *V. angularis* leaves under Cd stress (Figure 6 and Figure 7).

Abiotic stress can regulate the expression of genes involved in the capture of light energy, chlorophyll metabolism, and electron transport to regulate photosynthesis [13]. For example, the combined stress of mercury and water deficiency decreased the light-harvesting and electron transport capacity of maize by downregulating the levels of expression of the genes related to photosynthetic antenna proteins, photosynthesis, chlorophyll, and porphyrin metabolism, such as *PsbS1*, *PSBQ1*, and *FDX1*, which ultimately impaired its ability to conduct photosynthesis [66]. Salt stress significantly altered the photosynthetic capacity of tobacco (*Nicotiana benthamian*) leaves by downregulating a range of genes, particularly those related to the PSI reaction center (*PsaD*, *PsaF*, and *PsaG*), the PSII reaction center (*PsbO*, *PsbP*, *PsbQ*, and *Psb27*), the light-harvesting chlorophyll protein complex II (*Lhcb1*, *Lhcb3*, and *Lhcb4* among others), and the photosynthetic electron transport system (*PetE* and *PetF*) [67]. In this study, a total of 122 DEGs between the BR-Cd and Cd treatments related to chloroplasts and photosynthetic terms were screened using RNA-seq. In addition, the qRT-PCR results indicated that the BR treatment markedly enhanced the expression of the genes related to light energy capture, electron transport, and carbohydrate biosynthesis and metabolism under Cd stress. Overall, the BR treatment significantly enhanced the photosynthetic capacity of the *V. angularis* plant leaves under Cd stress and improved the carbohydrate metabolism cycle.

## 5. Conclusions

This study reveals how the BR treatment regulates the tolerance of *V. angularis* to Cd by controlling the antioxidant defenses, osmotic regulation, chlorophyll metabolism, and carbohydrate metabolism. The application of exogenous BR significantly increased the antioxidant content and enhanced the activity of antioxidant defense enzymes, which resulted in a decrease in the accumulation of ROS in the leaves of *V. angularis* seedlings. The BR treatment increased the amounts of osmotic regulatory compounds in the *V. angularis* leaves to maintain cellular homeostasis. The BR treatment promoted the biosynthesis, transformation, and degradation of chlorophyll under Cd stress, which increased the chlorophyll content and remodeling of the cellular structure. Furthermore, the BR treatment helped to alleviate the Cd damage to *V. angularis* by promoting the transcription of the genes related to photosynthesis and carbohydrate metabolism, thereby enhancing the accumulation of biomass. These results provide a reference for utilizing BR and its derivatives to regulate the growth of plants under Cd stress.

## Figures and Tables

**Table 1:** Effects of BR treatment on phenotype, biomass, and Cd accumulation of *V. angularis* under Cd stress.

Treatments	Plant Height (cm)	Leaf Area (cm^2^)	Leaf Dry Weight (g)	Above-Ground Dry Weight (g)	Growth Inhibition Ratio (%)	Cd Content (mg·g^−1^ DW)		
						Leaf	Stems	Petiole
W + Cd0	28.13 ± 1.10 ab	521.16 ± 28.47 a	1.41 ± 0.10 a	2.22 ± 0.15 a	-	-	-	-
W + Cd1	24.80 ± 1.93 cd	321.24 ± 15.94 c	0.79 ± 0.02 c	1.16 ± 0.02 c	38.38 ± 0.66 b	2.11 ± 0.33 b	1.13 ± 0.07 b	0.25 ± 0.01 ab
W + Cd2	22.43 ± 0.51 d	276.50 ± 11.40 d	0.77 ± 0.11 d	1.16 ± 0.10 d	47.62 ± 0.65 a	3.98 ± 0.25 a	1.68 ± 0.11 a	0.26 ± 0.01 a
BR + Cd0	29.77 ± 2.64 ab	506.69 ± 22.29 a	1.43 ± 0.08 a	2.21 ± 0.13 a	0.28 ± 0.11 d	-	-	-
BR + Cd1	27.57 ± 0.83 abc	435.56 ± 17.72 b	1.15 ± 0.06 b	1.75 ± 0.10 b	16.51 ± 0.23 c	1.68 ± 0.16 c	0.89 ± 0.09 c	0.21 ± 0.01 b
BR + Cd2	26.43 ± 0.81 bc	433.35 ± 20.35 b	1.16 ± 0.15 b	1.80 ± 0.17 b	16.89 ± 0.33 c	2.55 ± 0.13 b	1.13 ± 0.02 b	0.22 ± 0.02 b

The data presented represent the mean values ± standard deviations (SD) derived from three independent experimental replicates. Statistically significant differences (*p* < 0.05) among the various treatments are denoted by different letters. The same notation convention is adopted for all subsequent data points.

## Data Availability

The original contributions presented in this study are included in the article/Supplementary Material. Further inquiries can be directed to the corresponding authors.

## Supplementary Materials

The following supporting information can be downloaded at: https://www.mdpi.com/article/10.3390/biology14060674/s1, Table S1. Primers information.
