# Supplementary material for: 2,4-Epibrassinolide Mitigates Cd Stress by Enhancing Chloroplast Structural Remodeling and Chlorophyll Metabolism in Vigna angularis Leaves
Source: Biology (Basel). 2025 Jun 10;14(6):674. doi: 10.3390/biology14060674 (PMC12189853; doi:10.3390/biology14060674)
Supplement: Supplementary file 1 [file biology-14-00674-s001.zip › biology-3669345-supplementary.pdf]

Table S1 Primer information

Gene name	Gene ID	Forward primer (5'-3')	Reverse primer (5'-3')
VaHCF164	LOC108319169	CATGCATTCCCGTCCGAAGA	ACGAAAAGGTCACAAGAAGCAC
VaCLH1	LOC108345122	TCCCGTTGCTGTAATAGGCA	AGCACGGTCCCAATTATCCC
VaLHC3	LOC108319415	TCACCAAGCAGGTTTCCTCC	TTCCAACTCACGGTTCCTGG
VaLHC7	LOC108347462	TGGCAGTTTGCCTGGAGATT	AGCAGTGTACCATGAAGGGG
VaC-INV	LOC108323317	GACGGTGAGTCTGGTGACAG	AATGCTCTGCCTCCCTTTCC
VaBAM1	LOC108345955	GACGGTGAGTCTGGTGACAG	AATGCTCTGCCTCCCTTTCC
VaSuSy	LOC108331593	CAACCCACCACTCTTCACCA	ATGCGCCATATCTTCAGGGG
VaPAO	LOC108331598	CCCACTCGAACTTTCCTCCC	GCTTTTAAGTCCGCAGTGGC
VaPPH	LOC108335192	CTGAGGGATGCATACGGTGG	ACGTCATTGGGGGAAAGCAA
VaCAO	LOC108338340	GATGCATTGGAGCGAGGAGA	GAGTTCCCGGATGGTGTTGT
VaPASK	LOC108322634	CCTGTGCAGAACTTGGTTGC	TGTTCGCTGATGGAGCCAAT
VaHemY	LOC108329073	AGCTTCAACGTACTTTTGCTGT	ATGTTGGTTCCCCCTTTGCT
VaFNR2	LOC108343296	TTGACTTTGTCTCAGGCTGCT	GGTGCCCATGACTTATCCCG
VaPASN	LOC108334969	AACTCCAGTGTCTTGGCCTG	TTGAGAGCAGAAGCCTGTGG
VaHCR	LOC108326663	TGGTGTAGGTTGTCAGGTGC	TGGTGTAGGTTGTCAGGTGC
VaCHLH	LOC108334121	CCGCTCAAGTTAACGCTTCAG	CATTTCGTCCTGCCCCACTA
VaNYC1	LOC108346118	AGCCATGCGTGTGATGAGAA	TGGATCGCTTGCACTCCTTT
VaFNR3	LOC108341821	TCTCACCTGCGTTGCTTACC	AGGCAGCACACATGATGGAG
VaFNR4	LOC108343942	TCATCAAGGGGTTGGCTGAC	GCAGTGGCATTCCTTCCTCT
VaRCCR	LOC108337053	CCAGCGAGATGTTGGAGAGG	CCAGTACTCGGTTTGCCACT
VaSSS	LOC108342757	GCCATGTGGACTCACTCAACT	CAAGACCCTGTGCTTGTGCT
VaCHLE	LOC108329545	GTTTGTGGCCTTGCTTCAGG	ATTCAGCAGTGCAGGACCTC
VaPSBP4	LOC108330875	ATCGCAGGCCAGATGAGAAG	ATCGCAGGCCAGATGAGAAG
VaGluRS	LOC108326324	AGTGTTGTATCCGGGGCAAG	TTCACTTGATCGCAGGGCAT
VaCLH2	LOC108325428	GGAGTGGACCCAGTTGATGG	CCCTTAGGTGCACAAGGAGG
VaCHLG1	LOC108320403	GTGGGTGGAGCCTTACTGTC	TCCCAAACAAAGCCTGACCA
VaHemF	LOC108338699	TGCCCTTCACAGAGCATCAG	ATCCTGCCCCCAGTTTTCAG
VaFdC2	LOC108344331	GGTCGACGACAAAACCGGAA	CCTTCACCCACTCAGTAGCG
VaA-INV	LOC108336999	TGGGCTCACTCTGTGTCCTA	ATAGTGGCTGACCCTGACCA
VaDVR	LOC108341969	AGAGGAGCGGAATTAGGGGT	CTTGCAAGGCAAGACACCAC
VaHemL	LOC108331853	GCAGGCCATGCAATATGTGG	AGCCTCAAACTGGGATGGTG
VaSPS	LOC108329724	TGCAACTGCTCTTGAGGGAG	GCTGAAGCAAGCATCACGAG
VaHemB	LOC108331727	TAAAGGCTGCTGGTGCTCTC	CCACACAAACATCTGGCAGC
VaPSBW	LOC108325118	CAAGCAGCTCAAGCATGGTG	TGTTGCTAAGCCCAAAGGGA
VaHemC	LOC108320790	CATCAGACTCAACATTTCGCTGT	GCATTTGGGAGATGGCAACG
VaHemE	LOC108329525	CATGAGGCAAACAGGGAGAGT	CCTGGGCATTTCATCAGGCT
VaCHLG2	LOC108319129	GCATTGGTTAGGGTAGGACCA	TGCTAGCTCTGGATGCGATG
VaPETC	LOC108332507	CCCTTCTCAGCTATGCTCCG	GGCCTGGCAAGCAATTTTCA
VaHemD	LOC108341123	GCTTCTGGGATGCCTCATGT	AGCCTTTGCAGAAGCAGGAT
VaPETE	LOC108341895	CTTCGTTCCCAGCGACTTCT	CTTGGCACTCAAAGTGACGC
VaCHLM	LOC108331902	GGTTGGCTGGACCATAAGGA	CCTTGGTTCCGGAAGAGGAC

VaPASH6	LOC108333976	TGCTGGATCTTCCCTTGCTG	TGTAGGGTGAAGGTGCATCG
VaAMY1	LOC108343121	CGTGTTTGGATCTCGGGGAA	TCCAAGTGGATTCCGACAGC
VaFNR1	LOC108342331	TGATCACCCCAGAGGGAGAG	TCTGGTCCACTTTGCCAGAC
VaTUA3	LOC108329309	CATGCTGCCTGATGTATCGC	GGGCACCAGTCAACAAACTG
